# The old nourishes the new: 100 years of leaf senescence studies in *Plant Physiology*

**DOI:** 10.1093/plphys/kiag697

**Published:** 2026-09-21

**Authors:** Pablo Ignacio Calzadilla, Judy Brusslan

**Affiliations:** Institute for Integrative Biology of the Cell (I2BC), Université Paris-Saclay, CEA, CNRS, 1 Av. de la Terrasse Bâtiment 21, 91190 Gif-sur-Yvette, France; Department of Biological Sciences, California State University, Long Beach, 1250 Bellflower Blvd., Long Beach, CA 90840-9502, United States

## Abstract

The remarkable efficiency of plants is exemplified by leaf senescence, in which older leaves slowly perish while catabolizing and exporting the nutrients that made them prolific photosynthetic factories. Several endogenous (eg ontogeny and phenology) and exogenous (eg environmental stressors) factors can induce this physiological process. These multiple pathways, together with their complex regulatory networks, make senescence challenging to understand. The coordination between organelles, cells, and organs, across multiple levels of regulation (eg, genetic, metabolic, and physiological), further complicates the identification of the signals that initiate this process and the associated transduction pathways. Nevertheless, unraveling this complexity has remained a significant goal for plant biologists.

The critical process of leaf senescence has been studied in *Plant Physiology* for 100 years. Research has focused on 6 main questions: (i) Which hormones regulate leaf senescence? (ii) How is highly abundant Rubisco catabolized? (iii) How do reproductive sinks regulate leaf senescence? (iv) How does photosynthesis change during leaf senescence? (v) What role do reactive oxygen species (ROS) play in leaf senescence? and (vi) How does gene expression change during leaf senescence? This review focuses on *Plant Physiology*'s long-standing track record of publishing the foundational, field-defining work that has shaped core conceptual advances in leaf senescence research and provides context for future studies that will continue to address how leaves slowly and productively end their lives. Many other contributing papers have been cited in this review; however, our coverage is incomplete. We suggest readers view additional reviews for a more complete understanding of leaf senescence (Woo HR, Kim HJ, Lim PO, Nam HG. 2019. Leaf senescence: systems and dynamics aspects. Annu Rev Plant Biol. 70:347–376; Guo Y et al. 2021. Leaf senescence: progression, regulation, and application. Mol Hortic. 1:5; Zhang Y-M, Guo P, Xia X, Guo H, Li Z. 2021. Multiple layers of regulation on leaf senescence: new advances and perspectives. Front Plant Sci. 12:788996; Cao J, Liu H, Tan S, Li Z. 2023. Transcription factors-regulated leaf senescence: current knowledge, challenges and approaches. Int J Mol Sci. 24:9245; Zhai Y, Qiao W, Yang W, Liu X. 2026. Regulatory network of leaf senescence in Arabidopsis: the roles of transcription factors and microRNAs. Int J Mol Sci. 27:3584).

## Which hormones regulate leaf senescence?

Phytohormone regulation of senescence has often been studied in detached leaves, in which rapid senescence allows an amenable study system (Gan and Amasino 1997). Cytokinins (CKs) were first reported to delay yellowing of detached *Xanthium* leaves (Richmond and Lang 1957). Subsequent studies published in *Plant Physiology* established that CKs slow all measured aspects of dark-induced senescence in excised leaves of *Xanthium* (Osborne 1962) and barley (Srivastava and Ware 1965): sustaining the synthesis of chlorophyll, protein, RNA, and DNA, while decreasing DNase and RNase activity. Gibberellic Acid (GA) played a similar role in slowing chlorophyll, protein, and RNA loss (Beevers 1966). A decade later, CKs were reported to inhibit, in a dose-dependent manner, the increase in oxygen consumption seen 1 to 2 d after the start of dark treatment in both *Rumex* (Goldthwaite 1974) and oat (Tetley and Thimann 1974). A similar observation was noted also for GA (Goldthwaite and Laetsch 1968). A decrease in endogenous GAs was measured prior to leaf senescence in lettuce and pea (Aharoni and Richmond 1978; Zhu and Davies 1997) and a similar decrease for CKs was observed in soybean (Noodén et al. 1990).

In whole plants grown in the light, synthesis of endogenous CKs delays senescence, strengthening the role of this hormone as a negative regulator of leaf senescence (Gan and Amasino 1995). In this regard, transgenic lettuce plants expressing the isopentyl transferase gene (*IPT*, involved in CKs biosynthesis) downstream of the senescence-specific SAG12 promoter (*pSAG12-IPT*) were reported in *Plant Physiology* (McCabe et al. 2001). These lines specifically expressed *IPT* in senescent leaves, increasing CKs biosynthesis and, consequently, leading to the retention of protein and chlorophyll in older leaves. Higher levels of CKs increased sink strength of older leaves in the *pSAG12-IPT* plants. Thus, younger leaves of this line had lower protein and chlorophyll levels compared to the azygous controls. It should be noted that work supporting the uncoupling of chlorophyll and protein loss was also published in *Plant Physiology*. The nonyellowing (NY) meadow fescue mutant maintained high chlorophyll levels in detached leaves with dark treatment while protein decreased at a rate similar to the WT (Y) line (Thomas and Stoddart 1975). Thus, all aspects of senescence do not need to occur together.

A series of studies published in *Plant Physiology* during the 1970s also described abscisic acid (ABA) as a regulator of leaf senescence. ABA was found to accelerate RNA degradation and slow protein and RNA synthesis during dark treatment of detached *Rhoeo discolor* leaves (De Leo and Sacher 1970). The ABA promotion of dark-induced leaf senescence was partially reversed by CKs, and kinetic modeling suggested that the 2 hormones acted independently (Manos and Goldthwaite 1975). Now this observation is well accepted since the molecular signaling pathways of these hormones are understood. A surge in ABA levels correlated to chlorophyll degradation in dark-treated lettuce leaves (Aharoni and Richmond 1978). However, ABA levels increased in field-grown soybeans after the initial drop in chlorophyll and protein, suggesting ABA accelerated but did not induce leaf senescence under field conditions (Samet and Sinclair 1980). The onset of molecular genetics identified NAC transcription factors that were induced by ABA during leaf senescence, which promoted ABA synthesis (*NCED*) and transport (*ABCG40*) while inhibiting ABA inactivation (*ABA8ox*). Key transcription factors identified in *Plant Physiology* include AtNAP and ATAF1 in Arabidopsis (Zhang and Gan 2012; Garapati et al. 2015), OsNAC2 in rice (Mao et al. 2017), and SlNAP2 in tomato (Ma et al. 2018).

Additional studies in *Plant Physiology* focused on ethylene, which was shown to be a positive regulator of leaf senescence in dark-incubated excised lettuce leaves treated with Ethephon, an ethylene-releasing compound (Aharoni and Richmond 1978). Follow-up studies demonstrated that the ethylene-synthesis inhibitor AVG slowed chlorophyll loss (Aharoni et al. 1979a), while addition of ACC (promoting ethylene synthesis) accelerated this process (Gepstein and Thimann 1981). Additionally, a climacteric-like increase in both ethylene and respiration coincided with the rapid phase of chlorophyll loss (Aharoni et al. 1979b). Treating mature Arabidopsis rosettes with ethylene for 3 d could induce leaf senescence specifically in older rosette leaves. This response was completely blocked by a gain-of-function *SR1* allele. The SR1 calmodulin-binding transcription factor suppresses the key ethylene transcription factor gene, *EIN3,* and the main salicylic acid (SA) biosynthesis gene in Arabidopsis, *SID2* (Nie et al. 2012). The *SUB1A* gene, encoding an ERF transcription factor that confers submergence tolerance, also decreases ethylene synthesis in prolonged darkness, maintaining chlorophyll levels and slowing senescence (Fukao et al. 2012).

SA is another positive regulator of leaf senescence, which was shown to be required for reactive oxygen species (ROS)-induced senescence (Zhang et al. 2013). ROS induce the expression of the *WRKY75* gene, which encodes a transcription factor that directly targets and promotes *SID2* expression and SA biosynthesis, forming a feed-forward loop (Zhang et al. 2017). One of the most highly cited *Plant Physiology* papers describes an early transcriptome analysis comparing developmental and dark-induced senescence. High induction of *SID2* was only observed in developmental senescence, suggesting SA may not be involved in senescence of detached dark-treated or individually darkened leaves (van der Graaff et al. 2006).

In addition, the initial identification of the phytohormone jasmonic acid (JA) was published in *Plant Physiology* based on its action as a senescence promoter (Ueda and Kato 1980). Mutants in the JA receptor, COI, showed greatly reduced leaf yellowing (Shan et al. 2011). The *coi* mutants also had higher levels of Rubisco activase (RCA), suggesting that RCA is a COI-dependent JA-repressed protein, supporting a role for JA in reducing carbon fixation during leaf senescence. Auxin regulation of leaf senescence is complex, with both negative and positive regulation being supported by different studies. Some of these studies have been published in *Plant Physiolology*; however, a historical review of this topic requires publications in many journals.

In early work, exogenous addition of the naturally occurring auxin, indole-3-acetic acid (IAA), decreased *SAG12* expression (Noh and Amasino 1999), but free (unconjugated and active) IAA levels increased as senescence proceeded (Quirino et al. 1999). Mutants in the Auxin Response Factor2 (*arf2*) displayed delayed leaf senescence, but one study suggested this is due to a decrease in ethylene synthesis related to greatly reduced expression of *ACS2*, *ACS6,* and *ACS8* in *arf2* (Okushima et al. 2005). As mentioned above, ethylene is a positive regulator of leaf senescence. This simple explanation is contradicted by an additive delay in leaf senescence seen in *arf2ein2* double mutants (Ellis et al. 2005), indicating that the delay in leaf senescence observed in *arf2* is not solely mediated by reduction in ethylene signaling. Delayed leaf senescence in *arf2* was reversed by mutation in *ANT1*, an AP2/ERF transcription factor mutant that maintains meristem identity, suggesting that ARF2 may suppress *ANT* gene expression to promote leaf senescence (Feng et al. 2016). This is consistent with ARF2 suppressing expression of auxin-responsive genes (Tiwari et al. 2003). Direct support for a negative regulatory role is the strong reduction in the expression of many auxin transport genes in all forms of leaf senescence (van der Graaff et al. 2006). As leaf senescence proceeds, auxin transport is reduced.

Two other lines of inquiry address the role of auxin in the regulation of leaf senescence. One centers on the regulation of leaf senescence by a leucine-rich receptor (LRR)-kinase. The first functional validation of a receptor-like protein kinase/phosphatase pair involved in the regulation of leaf senescence, AtSARK and SSPP, respectively, was reported in *Plant Physiology* (Xu et al. 2011; Xiao et al. 2015). Overexpression of soybean SARK (GmSARK) in Arabidopsis signals early leaf senescence. This is reversed in *aux1* mutants and in seedlings treated with *p*-chlorophenoxyisobutyric acid (PCIB) (an auxin synthesis antagonist), suggesting both auxin transport and auxin synthesis are essential for GmSARK-mediated early leaf senescence (Xu et al. 2011). This supports a positive regulatory role for auxin.

SSPP, a 2C-type protein phosphatase, binds to and is regulated by Small Auxin Up RNA (SAUR) proteins (Wen et al. 2020). Expression of *SAUR* genes is rapidly upregulated by auxin and SAUR proteins are quickly degraded via the 26S proteosome (Hagen and Guilfoyle 2002). SAUR proteins have been shown to bind to and inhibit PP2C-D phosphatases, in this case activating H+-ATPases needed for apoplast acidification associated with auxin-induced cell elongation (Spartz et al. 2014). Overexpression of SAUR39, SAUR49, a stable version of SAUR36, SAUR72 and SAUR10 all resulted in early leaf senescence, and *saur36* mutants displayed delayed leaf senescence (Kant et al. 2009; Hou et al. 2013; Bemer et al. 2017). The mechanism by which SAUR promotes leaf senescence was demonstrated by SAUR49's ability to interact with SSPP in planta. The delay in leaf senescence mediated by SSPP overexpression was overcome by *SAUR49* overexpression, suggesting that SAUR49 interaction inhibited SSPP dephosphorylation of SARK, thus allowing leaf senescence to proceed (Wen et al. 2020). SAUR49 was found to be more stable in older leaves, and SSPP interaction with numerous other SAUR proteins was observed via yeast two-hybrid studies (Wen et al. 2020). Overall, these findings support a role for stabilization of some SAUR proteins in older leaves, which bind to SSPP, thereby promoting SARK-dependent leaf senescence. Importantly, how this regulatory system directly relates to auxin has not been established; indeed, some *SAUR* genes, including *SAUR49*, are not regulated by auxin (van Mourik et al. 2017). Notably, both *SAUR36* and *SAUR49* were identified as senescence-associated genes (SAGs) in a recent single-cell nuclear atlas of leaf senescence (Guo et al. 2025). Future focus on SAUR stabilization in older leaves would be a worthwhile pursuit.

Another line of inquiry focuses on the YUCCA proteins, which encode both flavin mono-oxygenases (FMO), which synthesize IAA, and thiol reductases (TR), regulating ROS homeostasis. Both YUC6 and YUC1 overexpression delayed senescence, supporting auxin as a negative regulator of leaf senescence (Kim et al. 2011). Further support for auxin's negative regulatory role was no delay in leaf senescence when YUC6 point mutants that inhibited NADPH cofactor binding, thereby thwarting FMO activity, were overexpressed. Additionally, the*YUC6* overexpression-mediated delay in leaf senescence was reversed by expression of an IAA-lysine synthetase (35S:iaaL, Kim et al. 2011). However, the role of YUC6 is more complex, as discovered by a point mutant (C85S) that inhibited the TR activity without inhibiting auxin synthesis (Cha et al. 2015). Lines that overexpress YUC6^C85S^ show more rapid leaf senescence due to increased ROS (Cha et al. 2016), suggesting that YUC proteins balance leaf senescence regulation via expression of auxin, a negative regulator, and ROS, a positive regulator.

Overall, a positive regulatory role promoting leaf senescence is supported by higher free IAA levels, *arf2ein2* additive double mutants, and *aux1* and PCIB reversal of GmSARK-induced leaf senescence. A negative regulatory role slowing leaf senescence is supported by exogenous IAA repressing *SAG12* expression, down-regulation of auxin transporter genes, and delayed senescence in *YUC6* overexpression lines being reversed by auxin conjugation to lysine. Multiomic approaches may help to disentangle these observations, allowing time-sensitive measurements of auxin metabolites, proteins and their phosphorylation status, and transcripts as leaves undergo senescence.

## How is highly abundant Rubisco catabolized?

Since ribulose bis-phosphate carboxylase/oxygenase (Rubisco) is the major reservoir of leaf nitrogen, a relevant question in the field of senescence has been the identification of the Rubisco-catabolizing proteases. This question remains unanswered despite 50 years of research. In an early 1970s *Plant Physiology* paper, Martin and Thimann added cycloheximide (a protein synthesis inhibitor) to detached dark-treated oat leaves and noted that protein degradation was prevented. This suggested the need for active protein synthesis for leaf senescence (Martin and Thimann 1972). In 1975, chloramphenicol, which inhibits chloroplast protein synthesis, was shown to have no effect on Rubisco loss in detached dark-treated barley leaves, narrowing the site of protein synthesis to the cytosol (Peterson and Huffaker 1975). In agreement, proteins were degraded slowly in purified oat senescing chloroplasts, suggesting limited chloroplast localized protein catabolism (Choe and Thimann 1975).

Many early papers also showed increased protease activity in cellular extracts concomitant with protein loss, which was suggested to be due to increased protease synthesis. The location and timing of protease activity was shown to correlate to leaf senescence in field-grown corn, with highest activity in the oldest leaves and in the later months of the growing season (Feller et al. 1977). Moreover, a serine-type endoprotease (pH optimum 4.4) and a sulfhydryl-type exoprotease (pH optimum 6.6) were purified 500-fold from intact dark-treated oat seedlings (Drivdahl and Thimann 1977, 1978). In these early papers, protease activity was assayed using hemoglobin or casein as substrates. Rubisco was first reported as a proteolytic substrate of a sulfhydryl-type protease by Wittenbach in 1979 when studying field-grown wheat. This sulfhydryl-type protease was determined to have a pH 4.8 to 5.0 optimum (Wittenbach 1979).

In the following decade, 3 other barley endoproteinases (EP1, EP2 and EP3), isolated from 12d-old light-grown leaves, were also reported to degrade Rubisco (Miller and Huffaker 1981, 1982, 1985). EP1 (28.3 kD) cleaved the large subunit; EP2 (67 kD) cleaved the small subunit; and EP3, which co-purified with Rubisco, was active on both subunits. The importance of EP1 and EP2 for Rubisco degradation was questioned when a large induction of these protease activities in dark-treated detached barley leaves did not parallel the rate of Rubisco loss (Miller and Huffaker 1985).

Subcellular partitioning of wheat mesophyll protoplasts showed vacuolar proteolytic activity for Rubisco. Accompanying ultrastructural studies showed chloroplasts overlapping the central vacuole in senescing leaves (Wittenbach et al. 1982). Mature forms of the RD21A papain-like cysteine protease accumulated in the vacuoles of older leaves (Yamada et al. 2001); however, subsequent work showed normal Rubisco loss in individually darkened leaves in *rd21a* mutants (Pružinská et al. 2017). *RD21B* and *RD21C* paralogs are expressed at lower levels than *RD21A,* and Rubisco retention during senescence has not yet been reported for triple *rd21* mutants. *SAG12* encodes a cysteine protease that is highly and specifically induced during leaf senescence; however, *sag12* mutants showed normal Rubisco degradation in individually darkened leaves (Pružinská et al. 2017). The identification of Rubisco-degrading enzymes has been difficult, in part, due to the redundancy of this important physiological function. CRISPR-Cas9 systems may be valuable in producing higher-order gene inactivation mutants. An alternative forward genetics approach might be the selection of mutants that retain GFP-tagged Rubisco during dark treatment of detached leaves. Proxiome studies of Rubisco in senescing leaves may also yield protease candidates; however, the tight interaction between the subunits will complicate these experiments.

Rubisco containing bodies (RCBs, Chiba et al. 2003) and Senescence Associated Vesicles (SAVs, Otegui et al. 2005) were hypothesized to transport Rubisco to the vacuole during senescence. In work published in *Plant Physiology*, RCBs were not detectable in autophagy mutants, but these mutants demonstrated normal Rubisco loss in individually darkened leaves. Thus, RCBs are not a required component for Rubisco catabolism (Wada et al. 2009). Loss-of-function SAV mutants have not yet been studied since the SAV protein components remain to be identified. After 50 years of research, with many important contributions published in *Plant Physiology*, Rubisco catabolism, the major source of recycled nitrogen, remains an open question.

## How do reproductive sinks regulate leaf senescence?

During senescence, once Rubisco and other proteins are catabolized, nutrients are recycled to the reproductive organs that act as sinks. The relationship between source (leaves) and sink organs in the regulation of leaf senescence has been a focus of study for more than 100 years. Within this journey, several relevant papers were published in *Plant Physiology*. In 1926, Murneek first reported that older tomato plants recover their vigor when defruited (Murneek 1926). Thirty years later, removing reproductive metabolic sinks and observing the effects on vegetative leaf senescence emerged as an active area of research. Early studies showed robust, green deflorated plants adjacent to florated control dead plants for both soybeans and spinach (Leopold et al. 1959). An elegant study aimed to define the source of the “senescence signal” using deseeding or sham operations on young soybean pods. These studies demonstrated that early deseeding prevented monocarpic senescence, suggesting the young seed as the source of the “senescence signal” (Lindoo and Noodén 1977). Although the “senescence signal” produced by seeds was not identified, later work published in *Plant Physiology* supported cytokinin as the “anti-senescence signal”, since zeatin riboside and dihydrozeatin increase dramatically in xylem root sap collected from depodded soybean plants (Noodén et al. 1990).

In the 1980's, depodding experiments gained in sophistication, and a more complex picture of the leaves that remained green after pod removal emerged. In both greenhouse and field-grown depodded soybeans, chlorophyll was retained, and starch increased, yet Rubisco levels and photosynthesis (CO_2_ assimilation) decreased. Interestingly, 3 proteins (∼27, 29 and 80 kD) accumulated abundantly in the paraveinal mesophyll cells of green leaves of depodded soybean plants, as visualized by SDS-PAGE. Antisera raised against these proteins suggested they were vacuolar glycoproteins that played a role in nitrogen storage and leaf longevity, allowing eventual reproduction (Wittenbach 1982, 1983). Further work showed similar induction of these glycoproteins in different soybean cultivars with differing rates of Rubisco retention, suggesting these proteins played a central role in maintaining the viability of the leaves with varying rates of photosynthetic decline (Crafts-Brandner and Egli, 1987).

The genes encoding the 27 kD and 29 kD proteins were reported in a 1988 *Plant Physiology* paper and named vegetative storage proteins (VSP). The strategy for cloning required in vitro translation of size-selected mRNA and antibody binding to identify the correct cDNA clones (Staswick 1988). A third VSP clone for the 94 kD protein (VSP94) was identified in 1991 and encoded a lipoxygenase that localized to the paraveinal mesophyll cells (Tranbarger et al. 1991). All 3 *VSP* transcripts were upregulated in depodded soybean leaves. Arabidopsis orthologs have been identified: VSP27: AT5G24770 (*VSP2*), VSP25: AT5G24780 (*VSP1*) and AT1G55020 (*LOX1*). VSP2 acts as an anti-insect phosphatase (Liu et al. 2005), while a defense role for *LOX1* is suggested by its up-regulation by pathogens and its role in oxylipin biosynthesis (Melan et al. 1993).CRISPR-Cas9 can now be used to inactivate the soybean genes to provide genetic support for their role in nitrogen storage in older leaves.

In maize, removal of young ears produced strikingly different results than depodding in soybeans. Leaves above the removed ears turned red and showed a rapid loss of chlorophyll, accompanied by increased sugar and starch accumulation (Allison and Weinmann 1970). The responses to ear removal were studied in leaves and stalks a decade later (Christensen et al. 1981). As before, leaves in de-eared plants initially retained carbohydrates and showed a more rapid chlorophyll loss accompanied by reduced nitrogen accumulation. In contrast, dramatic increases in the levels of stalk carbohydrates and nitrogen suggested that the stalk becomes a storage organ when ears were removed. Forty years later, pollination was prevented in maize, and the resulting transcriptome of senescing leaves shared many genes with sugar-induced senescence in Arabidopsis (Sekhon et al. 2012). In addition, an increase in fructose and glucose was observed in a metabolic study of age-induced plant senescence in Arabidopsis (Watanabe et al. 2013). These parallel studies in soybeans and maize suggest plants use different strategies to store nutrients to maintain longevity if the reproductive sink is removed.

The movement of nitrogen from source to sink was studied in 2 highly cited *Plant Physiology* papers. In source leaves, glutamate serves as a substrate for glutamate dehydrogenase, releasing ammonia that is assimilated into glutamine by the enzyme glutamine synthetase (GS) in both the chloroplast and the cytosol (GS2 and GS1, respectively) (Masclaux-Daubresse et al. 2006). Transport of nitrogen from source to sink was measured by^15^N tracing experiments in which roots took up labeled nitrogen, which was incorporated into rosette leaves prior to the chase. During the chase, ^15^N moved from the rosette leaves to cauline leaves, siliques and flowers (Diaz et al. 2008).

In recent decades, research in this field has increasingly focused on understanding the molecular mechanisms through which sources and sinks communicate, and the signals that mediate this communication. In 1997, Jang et al. reported that the enzyme hexokinase (HXK)- involved in the hexose assimilation pathway—mediates sugar responses in plants (Jang et al. 1997). Later, its participation in several physiological processes was demonstrated, including leaf senescence (Dai et al. 1999; Moore et al. 2003; Zheng et al. 2021). Moore et al. (2003) further showed that HXK signaling capacity is independent of its catalytic activity, highlighting its dual role in sugar metabolism and signaling.

Beyond sugar sensing, plants also possess energy-sensing mechanisms that integrate the cellular metabolic status with developmental responses. In this regard, the protein kinase SnRK1 is an energy-sensing node involved in maintaining metabolic homeostasis in response to environmental stimuli and developmental cues (reviewed by Wurzinger et al. 2018; Wu et al. 2025). In a paper published in *Plant Physiology*, Cho et al., (2012) reported that overexpressing snRKA1 from rice delayed leaf senescence in Arabidopsis. Recently, snRK1 has been described as a key node in the source-sink communication for nitrogen remobilization (Pang et al. 2026).

Trehalose-6-phosphate (T6P), a phosphorylated intermediate of trehalose biosynthesis, is another important factor in the sugar-mediated senescence pathways (Wingler et al. 2012). Remarkably, a series of papers published in *Plant Physiology* established T6P as a key regulator of developmental responses through its inhibition of SnRK1 activity (Zhang et al. 2009a; Debast et al. 2011; Delatte et al. 2011; Martínez-Barajas et al. 2011). Within this context, the T6P/SnRK1 pathway has been implicated in resource allocation at the whole-plant level (Nunes et al. 2013; Oszvald et al. 2018). For a recent revision of the role of T6P in plant development, see SharathKumar et al. (2026). Recently, cytosolic arginine was shown to negatively correlate with leaf senescence and was proposed to be the beacon nitrogen molecule that signals that nitrogen mobilization is complete and senescence can be finished (Hussain et al. 2026).

Long-distance systemic signals, such as mobile peptides and RNA, can mediate source-sink communication in plants (Kondhare et al. 2025). These systemic signals regulate translation and several developmental processes (eg flowering, tuber formation; Corbesier et al., 2007; Tamaki et al. 2007; Banerjee et al. 2009; Zhang et al. 2009b). Recently, the long noncoding RNA *ELENA1* (Cheng et al. 2023) and several small CLAVATA3/ESR-RELATED (CLE) secreted peptides (Zhang et al., 2022a, 2022b; Zhao et al., 2025) and SCOOP peptides (Zhang et al., 2024) have been implicated in modulating leaf senescence. Their crosstalk between hormone signaling, ROS and organ-to-organ communication shed light onto the complexity of source-sink relationships in the regulation of senescence.

## How does photosynthesis change in leaf senescence?

The relationship between photosynthetic light reactions and leaf senescence has long been established. In a paper published in *Plant Physiology*, Goldthwaite and Laetsch (1967) demonstrated that DCMU (3-(3,4-dichilorophenyl)-1, I-dimethylurea), a PSII acceptor side inhibitor, accelerated senescence in *Phaseolus vulgaris* leaf discs exposed to light. However, Haber et al. (1969) and, later, Thimann et al. (1977) challenged this idea, showing that light delayed leaf senescence even in the presence of DCMU. Although the DCMU concentrations used were comparable between studies, the contrasting results may be attributed to differences in the length of light exposure or intensities applied. Accordingly, Hidema et al. (1991) showed that senescence progression is light-intensity dependent in rice, suggesting the activation of distinct molecular processes in each light intensity condition. High light can trigger alternative electron transport pathways in the chloroplast thylakoid membranes. In this context, Thimann et al. (1977) proposed that the light-dependent slowing of leaf senescence is mediated by ATP photoproduction through cyclic photophosphorylation. This process requires cyclic electron flow (CEF) around PSI and is not inhibited by DCMU.

CEF is an alternative electron pathway that recycles electrons from PSI back to the plastoquinone (PQ) pool, generating a proton motive force for ATP synthesis without producing reducing equivalents (NADPH) (Joliot and Joliot 2005). CEF balances the ATP/NADPH ratio under limited NADPH oxidation conditions. Moreover, it enhances photoprotection by promoting nonphotochemical quenching (NPQ) and the regulation of the cytochrome *b_6_f* (Cyt *b_6_f*) complex through the generation of a steep thylakoid ΔpH (Nawrocki et al. 2019). Two independent pathways of CEF have been proposed: a pathway dependent on the activity of the chloroplast NADH dehydrogenase-like (NDH) complex (Shikanai et al. 1998) and the PGR5/PGRL1 pathway (Munekage et al. 2002; DalCorso et al. 2008). The precise molecular actors and mechanisms of the PGR5/PGRL1 pathway are still a matter of debate.

During the 1980s, indirect evidence published in *Plant Physiology* pointed to a possible involvement of CEF in leaf senescence. First, stromal photosynthetic enzymes declined earlier than electron transport in senescent wheat leaves, while electron transport remained coupled to proton gradient generation (Camp et al. 1982). A decrease in carbon metabolism resulting from Rubisco protein degradation could promote CEF to adjust the ATP/NADPH ratio to the metabolic demand (Walker et al. 2014). Loss of Rubisco leads to lower NADPH consumption by the Calvin Benson Bassham cycle, while ATP is still required to fulfil the higher energetic demand of senescence (Malik and Thimann 1980). Second, an uncoupling of electron transport between PSII and the Cyt *b_6_f* complex has been reported during heat-stress–induced senescence in wheat cultivars (Harding et al. 1990). Third, the Cyt *b_6_f* complex has been proposed as the limiting step in the rate of electron flow in senescent chloroplasts (Ben-David et al. 1983; Holloway et al. 1983).

## What role do reactive oxygen species (ROS) play in leaf senescence?

ROS are oxygen-containing molecules produced as byproducts of cellular metabolism. In plants, chloroplasts are a major site for ROS production under illumination due to the presence of light-absorbing pigments (ie chlorophylls). Under certain metabolic conditions (eg high light, low CO_2_ availability, stress), the capacity for light energy utilization becomes limiting, resulting in over-reduction of the photosynthetic electron transport chain and increased electron transfer to molecular oxygen, which generates ROS. During senescence, Rubisco degradation can lead to the decline of carbon fixation, triggering limitation of downstream electron acceptors (eg NADP^+^). Under these conditions, the imbalance between the excitation energy absorbed and its consumption can result in the production of O_2_^.−^ and ^1^O_2_. In this regard, activation of CEF could also contribute to the redox poise by reducing electron transfer to oxygen (Murchie and Niyogi 2011).

With the advent of molecular biology, new evidence supports a link between photosynthetic electron transport and leaf senescence (Zapata et al. 2005; Wang et al. 2015; Mayta et al. 2018). A mutant of *Nicotiana tabacum* lacking the NDH F subunit, *ndhf,* showed delayed senescence and lower oxidative damage symptoms compared to the wildtype plant (Zapata et al. 2005). NDH levels and activity increase during leaf senescence in different plant species, supporting the involvement of NDH in this process (Zapata et al. 2005; Tallón and Quiles 2007). Nevertheless, similar mutations inhibiting assembly of the NDH complex in *Arabidopsis thaliana* did not result in alterations of leaf senescence (Wang et al. 2015). By contrast, mutations in STN7 and STN8 kinases, related to light acclimation, accelerated leaf senescence. Interestingly, STN8 was also linked to PGR5/PGRL1-dependent CEF regulation (Reiland et al. 2011). However, to the best of our knowledge, a causal connection between CEF and leaf senescence has not yet been demonstrated.

Altogether, well-established evidence links photosynthetic electron transport with leaf senescence. However, further research is needed to address: (i) How dismantling of the photosynthetic electron transport chain is regulated? (ii) Which are the first photosynthetic complexes to be degraded? (iii) Is alternative electron transport a regulatory node for leaf senescence or simply a downstream consequence of its progression? The regulatory pathways bridging photosynthetic electron transport and senescence might be related to (i) the need for ATP synthesis under conditions where linear electron flow is impaired, but energy is still required for dismantling the photosynthetic machinery and (ii) the production of photosynthetic-derived ROS that participate as signaling molecules during leaf senescence. For a more detailed discussion on this topic please refer to the following reviews Krieger-Liszkay et al. (2019); Mayta et al. (2019); Domínguez and Cejudo (2021).

Excess ROS accumulation produces oxidative damage to cellular components and promotes cell death (Scandalios 1993). In a foundational paper published in *Plant Physiology*, Jones and Kok (1966) showed how excessive light absorbed by photosynthetic pigments can damage photosystems and impair chloroplast photosynthesis, even before ROS were identified as the culprits. These observations set the basis for later work connecting oxidative stress with chloroplast degradation, particularly membrane damage and photoinhibition (Chia et al. 1981; Pauls and Thompson 1984).

Oxidative stress has been described as an intrinsic feature of senescence, with redox homeostasis playing a central role in the progression of leaf senescence (Thompson et al. 1987; del Río et al., 1998). In this regard, antioxidant enzymes and cellular redox buffers (eg ascorbate and glutathione) participate in redox poise, leading to senescence regulation (Jiménez et al. 1998). Accordingly, increases (or decreases) in ascorbate content have been observed to delay (or accelerate) the progression of senescence (Barth et al. 2004; Chen and Gallie 2006; Kotchoni et al. 2009; Chen et al. 2021). Serotonin was also identified in a *Plant Physiology* paper as a protective antioxidant expressed during leaf senescence in rice, with serotonin-overproducing lines exhibiting delayed senescence (Kang et al. 2009). Although ROS have historically been associated with senescence due to their damaging effects, evidence emerged in the late 1990s suggesting their function as signaling molecules in plants (Foyer et al. 1997; Wu et al. 1997; Potikha et al. 1999). In this context, the role of redox signaling networks in chloroplast dismantling and leaf senescence has also been reconsidered (Rogers and Munné-Bosch 2016).

Microarray analyses have significantly advanced functional studies on leaf senescence, reshaping our understanding of the role of redox regulation in this process (Guo et al. 2004; Buchanan-Wollaston et al. 2005; van der Graaff et al. 2006). In a manuscript published in *Plant Physiology*, Gadjev et al. (2006) performed a transcriptomic analysis of gene expression changes triggered by ROS in *Arabidopsis thaliana*. The authors identified thousands of genes whose expression was significantly altered in response to ROS, spanning multiple physiological processes. It was found that approximately one third of the transcription factors identified in *Arabidopsis* were responsive to ROS. Among them, WRKY6 was previously highlighted as being strongly associated with leaf senescence (Robatzek and Somssich 2001). Later, this connection was strengthened in a study of dark-induced senescence through transcriptomic profiling (Rosenwasser et al. 2011).

Other transcription factors (TF) whose expression is induced by ROS have also been implicated in the progression of senescence, such as *WRKY53*, which is induced by H_2_O_2_ (Miao et al. 2004). ATAF1 also functions as an H_2_O_2_-responsive transcription factor that coordinates chloroplast degradation and leaf senescence by regulating genes involved in cell death, photosynthesis, and ABA metabolism (Garapati et al. 2015). Further support for ROS signaling in leaf senescence has been provided by the development of bioinformatic tools designed to analyze ROS-related transcriptomic signatures (Rosenwasser et al. 2013). This study highlighted the contribution of mitochondrial stress to the early stages of leaf senescence. Subsequently, Chrobok et al. (2016) reinforced the involvement of mitochondria in this process by emphasizing their central energetic and metabolic roles in cell catabolism, including ATP and glutamate synthesis.

Altogether, evidence supports a dual role for ROS in leaf senescence, acting both as mediators of cellular degradation (Pauls and Thompson 1984) and as signaling molecules (Garapati et al. 2015; Chrobok et al. 2016). This dual function might depend on multiple factors, including their migration capacity, the time window in which they are produced, their subcellular localization, and the specific mechanisms that trigger their generation (Noctor and Foyer 2016). Although characterizing ROS dynamics and functions in vivo is technically challenging, recent studies in *Plant Physiology* have highlighted advances in genetically encoded ROS-detecting fluorescence biosensors (Dietz et al. 2016; Müller-Schüssele et al. 2021; Ugalde et al., 2021; Arnaud et al. 2023). The use of these genetic tools to study redox cellular dynamics will more precisely define the role of ROS during leaf senescence.

## How does gene expression change during leaf senescence?

The onset of the molecular biology era allowed for the identification of the senescence-associated genes (SAGs), which change their expression due to the initiation and/or progression of leaf senescence (Gan and Amasino 1997). SAGs encode nucleases (eg BFN1, Pérez-Amador et al. 2000), proteases (eg MMPs, Azumi and Watanabe 1991; Delorme et al. 2000), kinases and phosphatases (Hajouj et al. 2000; Zhou et al. 2009; Xu et al. 2011; Xiao et al. 2015), and enzymes generally involved in the degradation of the chloroplast and the photosynthetic apparatus (Jiang et al. 1993).

SAG transcription is regulated in a multifactorial way, being at the crossroads of hormones and ROS, nutrient availability, and stress (Schippers et al. 2015). In this regard, Miller et al. (1999) characterized the senescence-related gene expression induced by ozone in Arabidopsis plants and discussed how this response related to developmentally induced senescence. As in developmental senescence, ozone triggers accelerated downregulation of Rubisco small subunit genes (*rbcS*) and chlorophyll *a*/*b* binding protein genes (*cab*), while inducing the early expression of senescence-molecular markers such as *cch* and *bcb* (proteins related to metal remobilization). However, other known markers of leaf senescence (ie *SAG12* or *MT1*) were not induced by ozone treatment, highlighting that stress-induced senescence might not share the same signaling pathways as age-induced senescence.

During the early 2000s, He et al. (2001) designed Arabidopsis trap lines for the identification and analysis of novel SAGs. In this *Plant Physiology* paper, the authors transformed and screened more than 1,000 lines of Arabidopsis expressing the β-glucuronidase (GUS) reporter gene at different genome locations, aiming to identify senescence-specific GUS expression. Using this strategy, 127 specific senescence genes were identified and shown to be modulated by several regulatory pathways (eg ethylene, darkness, or drought). Later, using 2 cDNA libraries from the tree *Populus tremula*, Bhalerao et al. (2003) studied the genes expressed during autumn in leaves with no visible senescent symptoms against fully expanded young leaves. Cysteine proteases and metallothioneins were found to be upregulated in autumn leaves, together with a strong downregulation of plastid-related genes, pointing to the initiation of chloroplast degradation early during autumn senescence in *Populus*. Metallothioneins were previously reported to be upregulated during senescence in *Brassica napus* (Buchanan-Wollaston 1994).

Beyond the identification of SAGs, subsequent studies focused on uncovering the regulatory networks that coordinate their expression. Senescence genetic networks were explored by van der Graaff et al. (2006) and Rosenwasser et al. (2011), when comparing developmental and dark-induced senescence; and by Price et al. (2008) through comparisons of senescence across different plant organs. Later, Woo et al., (2016) compared changes in regulatory gene expression and in the abundance of different RNA types (eg mRNAs, siRNAs, ncRNAs) throughout leaf senescence and across cellular organelles. Brusslan et al. (2015) pointed to a higher level of senescence regulation through epigenetics, by characterizing the genome-wide abundance of 2 histone modifications (H3K4me3 and H3K9ac) during this process in Arabidopsis. These studies highlight the complexity of senescence and its regulation at different scales.

The increase in omics-related data sets and the advancement of high-throughput genetic technologies paved the way to the adoption of systems biology for studying leaf senescence (Kim et al. 2016; Skirycz and Fernie 2022). In this regard, a multiomics approach combining transcriptomics and metabolomics shed light onto the involvement of the cytochrome P450 gene *KLU* in cytokinin signalling and leaf senescence in Arabidopsis (Jiang et al. 2021). Recently, by performing time-course metabolomic and transcriptomic analyses, Yuan et al. (2026) identified candidate genes participating in the carbon source-sink regulation of leaf senescence in maize. In the upcoming years, this complex analysis of big datasets will be facilitated with the improvement of computational tools and the judicious use of artificial intelligence (Lam et al. 2024).

With the advent of multiomics studies and computational tools that synthesize data sets, it can be easy to forget the fundamental studies that helped plant biologists define the regulation and implementation of leaf senescence (Fig. 1). These early studies, a great proportion published in *Plant Physiology*, identified hormones and SAGs that promote and inhibit leaf senescence. Yet, many important questions remain unanswered: How is Rubisco catabolized? How do sinks communicate with senescing leaves? How is photosynthetic decline regulated during senescence, and what are its consequences for electron transport and signaling? How are ROS signals perceived during senescence? Here, we have provided a historical perspective on how *Plant Physiology* has contributed to shaping this research field. Reading these older studies has uncovered buried gems that provide value for the future century of leaf senescence studies. Indeed, the old nourishes the new.

**Figure 1:**
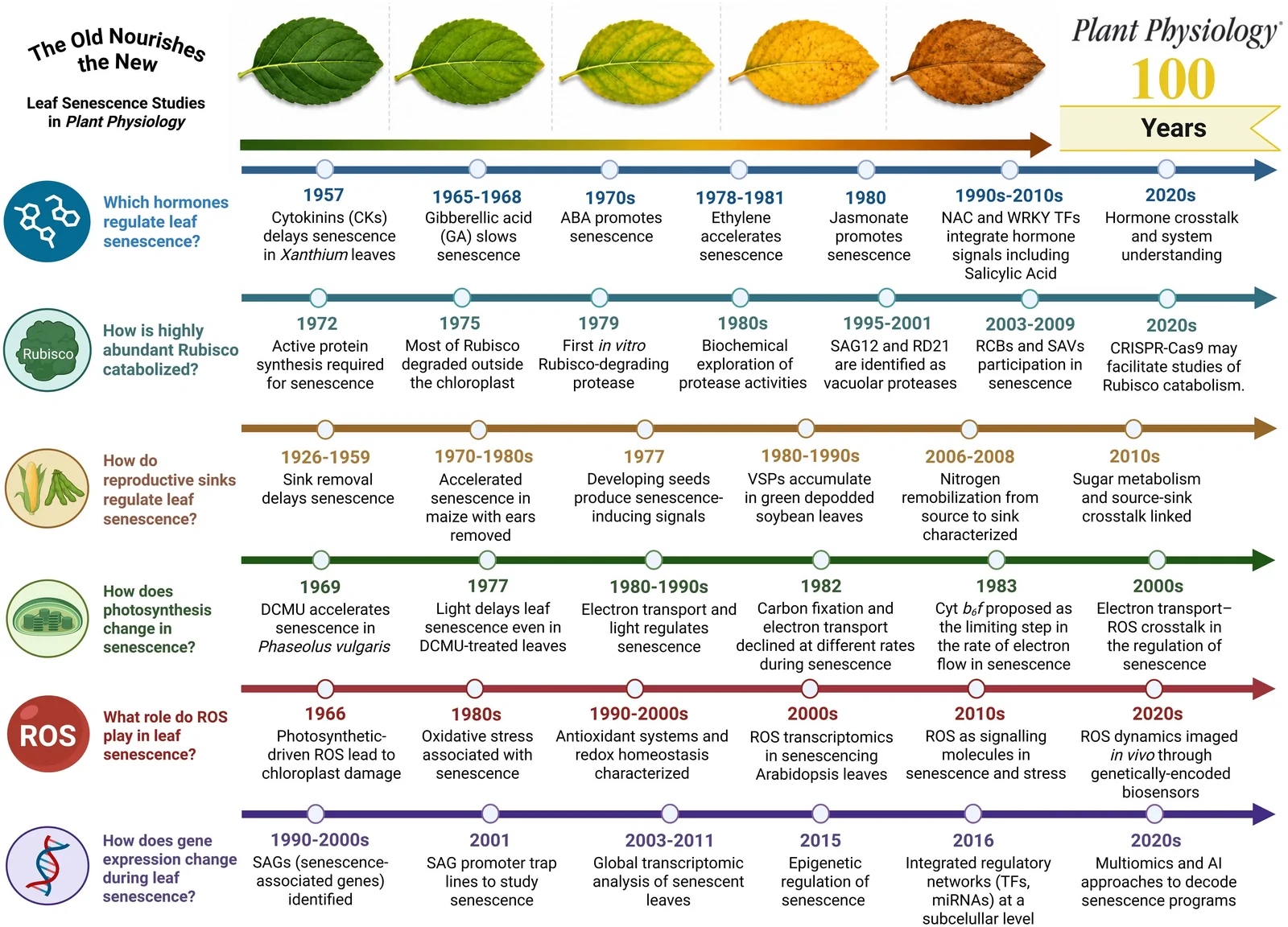
A century of discoveries in *Plant Physiology* illuminating the physiological and molecular dimensions of leaf senescence. Created in BioRender. Calzadilla, P. (2026) https://BioRender.com/8naclwb.

## Data Availability

No new data were generated or analysed in support of this research.
